# Right atrium crossover

**DOI:** 10.1093/ehjcr/ytad642

**Published:** 2023-12-26

**Authors:** Joana Brito, Rui Plácido, Pedro Matos

**Affiliations:** Department of Cardiology, Centro Hospitalar Universitário Lisboa Norte (CCUL@RISE), Faculdade de Medicina da Universidade de Lisboa, Av. Prof. Egas Moniz 1649-028, Lisboa, Portugal; Department of Cardiology, Centro Hospitalar Universitário Lisboa Norte (CCUL@RISE), Faculdade de Medicina da Universidade de Lisboa, Av. Prof. Egas Moniz 1649-028, Lisboa, Portugal; Department of Cardiology, Hospital CUF Tejo, Lisboa, Portugal; Department of Cardiology, Hospital CUF Tejo, Lisboa, Portugal

**Figure:**
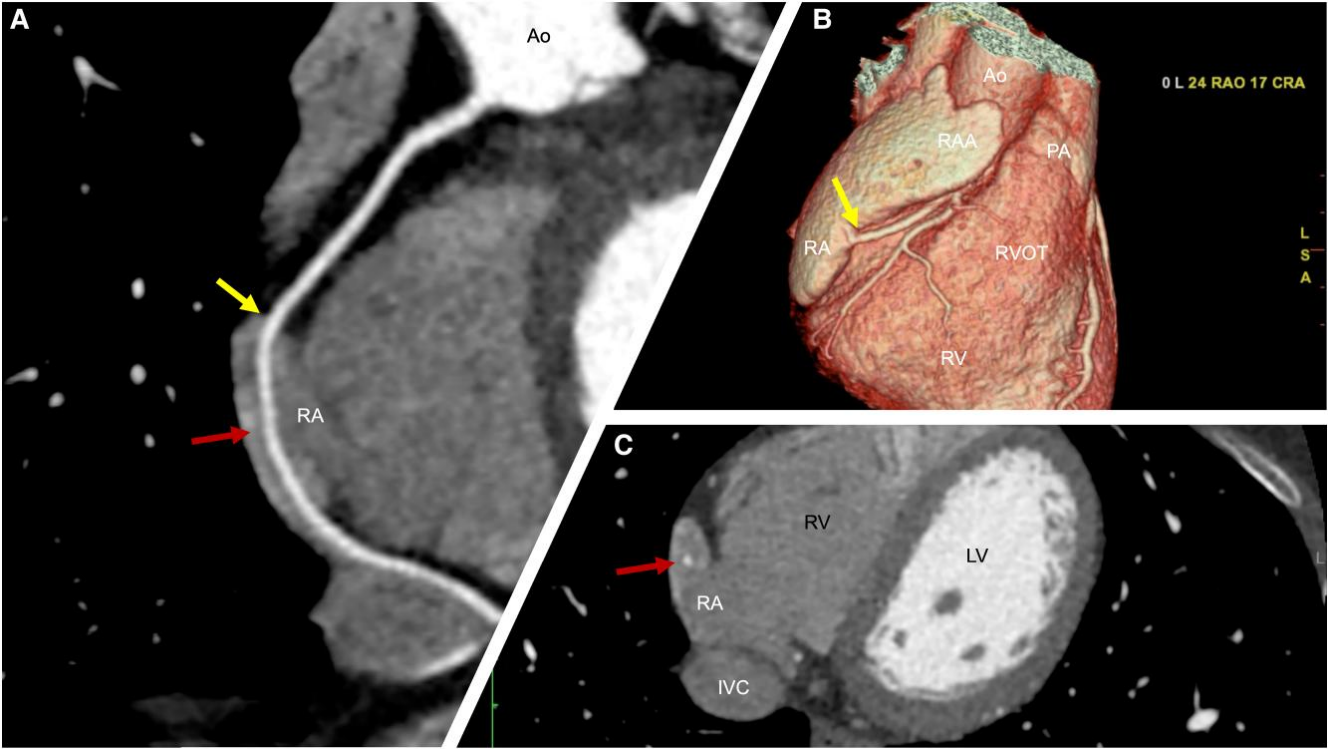


A 48-year-old woman with no relevant past medical history was referred for coronary computed tomography angiography (CCTA) due to complaints of atypical angina lasting for 2 months.

The CCTA depicted a coronary calcium score of 0 and no atherosclerotic disease. However, during the assessment of the coronary anatomy, an abnormal right coronary artery (RCA) course was observed (*Panels A, B, and C*), showing a proximal-to-mid segment with a high trajectory, above the atrioventricular groove (*Panels A and B*—yellow arrows). This was followed by an intracavitary course (*Panels A and C*—red arrows), entering the right atrium (RA) through the lateral wall, and exiting the cavity in the posterior wall, rejoining the right atrioventricular groove (see Supplementary material online, *Video S1*).

Intra-atrial RCA has been reported with an estimated incidence of 0.1–0.4%. This rare congenital coronary anomaly is particularly important in patients undergoing invasive procedures, namely, device implantation or electrophysiological procedures which are normally performed through the RA and may cause inadvertent traumatic lesion. Additionally, the potential risk of RCA injury during coronary bypass surgery has been described.

CCTA is a non-invasive diagnostic test method with a high ability to accurately evaluate coronary anatomy and safely exclude obstructive coronary disease. This case illustrates the importance of conducting a careful analysis of coronary courses in routine CCTA.

## Supplementary Material

ytad642_Supplementary_Data

## Data Availability

The data underlying this article are available in the article and in its online Supplementary material.

## Supplementary material

**Consent:** The author/s confirms that written consent for submission and publication of this case report including image(s) and associated text has been obtained from the patient in line with COPE guidance.

**Funding:** Nothing to declare.

